# The association between airborne pollen monitoring and sensitization in the hot desert climate

**DOI:** 10.1186/s13601-020-00339-6

**Published:** 2020-08-10

**Authors:** Maryam A. Al-Nesf, Dorra Gharbi, Hassan M. Mobayed, Blessing Reena Dason, Ramzy Mohammed Ali, Salma Taha, Amjad Tuffaha, Mehdi Adeli, Hisham A. Sattar, Maria del Mar Trigo

**Affiliations:** 1 Allergy and Immunology Section, Department of Medicine, Hamad Medical Corporation, Doha 3050, Qatar; 2 Pulmonary Division, Department of Medicine, Hamad Medical Corporation, Doha 3050, Qatar; 3 Sidra Medicine, Doha 26999, Qatar; 4 Department of Plant Biology, University of Malaga, Campus de Teatinos, Malaga, Spain

**Keywords:** Qatar, Allergy, Aerobiology, Pollen, Skin prick test

## Abstract

**Background:**

Pollen is a major cause of allergic respiratory diseases. In Qatar, data on the presence and prevalence of allergenic airborne types of pollen is quite limited.

**Methods:**

The study aimed to determine and correlate the most frequently implicated airborne pollen detected by aerobiological monitoring samplers in respiratory allergy symptoms. An aerobiological survey was started on May 8, 2017. Airborne pollen was collected using two Hirst type seven-day recorder volumetric traps. Skin prick test in patients attending allergy clinics in Doha using commercial extracts was conducted.

**Results:**

Twenty-five pollen types representing the native, as well as the introduced plants, with a relatively low daily mean concentration were observed from May 2017 to May 2019. The highest pollen concentrations were reached by Amaranthaceae (58.9%), followed by Poaceae (21.7%). SPT revealed a comparatively higher degree of sensitization to pollen. Among 940 patients, 204 were sensitized to pollen (54% female) with 135 (66.2%) and 114 (55.8%) to Amaranthaceae and Poaceae, respectively. Some patients had polysensitization. There was a statistically significant association between Amaranthaceae, and asthma (r = 0.169, *P *= 0.016) and allergic rhinitis (r = 0.177, *P *= 0.012).

**Conclusions:**

This is the first study to monitor airborne pollen in the state of Qatar. The main pollen detected were Amaranthaceae and Poaceae. Pollen may represent a possible exacerbating factor in adult patients with allergic diseases such as asthma and allergic rhinitis.

## Introduction

The prevalence of asthma and allergic rhinitis is high and increasing in western countries [1]. However, relatively little is known about the prevalence rate of allergic disorders in the Middle East and Arab Gulf countries. Specifically, the Qatari population has a high prevalence of diagnosed asthma (19.8%), allergic rhinitis (30.5%), and atopic dermatitis (22.5%) in children and adults [2].

Allergic diseases related to pollen are called pollinosis and include rhinitis, conjunctivitis, and asthma. During the last few decades, pollen has received increasing attention due to its strong allergenic potential, with severe impacts on human health [3]. There is a body of evidence suggesting that the prevalence of respiratory allergic reactions induced by pollen has been increasing in the most developed countries, especially in North America and Europe [3, 4].Understanding pollen emission dynamics is fundamental for the characterization of potential allergens that may be of greater health relevance in both natural and inhabited areas [5]. The allergenic content of the atmosphere varies according to climate, geography, and vegetation. The biological particles present in the atmosphere vary from place to place and from day to day. They mainly depend on the season, the type of vegetation growing in the surrounding places, plant phenology, and meteorological conditions [6].

Airborne pollen monitoring has become a standard practice in many countries [7]. During the flowering season, daily results and forecasts regarding types and quantities of allergenic airborne pollen and spores are reported. Pollen monitoring has helped in the answering of an array of different research questions, ranging from pollination biology to hay fever studies [8]. However, data in the Arabian Gulf are scarce [9]. Among the countries in this region, Kuwait was the origin of many investigations describing the seasonal distribution of airborne pollen [10, 11], followed by Saudi Arabia [12, 13]. All the previous studies were conducted by using the Hirst type volumetric spore trap.

To date, a few studies have been conducted in Qatar in order to determine and characterize sensitization to most common airborne pollen types and inhalant allergens. One-thousand one-hundred and six patients with clinical signs of allergy, attending a clinic at a tertiary hospital in the capital city of Doha, were evaluated [14, 15]. 51.4% had at least one positive skin prick test (SPT). The most common sensitizations were to Dermatophagoides pteronyssinus (Der p l) (41.6% of the patients), Dermatophagoides farinae (Der f l) (36.9%), Cockroach (Bla g l) (32.2%), and Chenopodium (13.6%) [14]. Furthermore, out of 134 children attending an allergy clinic in Doha, 19.4% were sensitized to at least one aeroallergen. The most frequent sensitizations were to allergens in the cat dander (16.4%), dust (14.9%), dog dander (11.9%), and ryegrass (2.2%) [15]. However, no aerobiology data were reported using volumetric samplers. Additionally, an accumulating body of evidence indicates that both, lifestyle factors in Qatar and environmental exposure, may play particularly important roles in the occurrence of respiratory symptoms as reported in a study showing a significant relationship between pet ownership and respiratory allergy [16]. Urbanization, pollution, and possibly imported plants that are factors arising from lifestyle changes may potentially have an impact on humans health and maintaining a healthy ecosystem [17].

The undertaken study was conducted to estimate the prevalence of common pollen sensitization and allergic respiratory diseases among adult patients attending allergy clinics in a tertiary hospital in the state of Qatar; a country with hot desert climate, and to investigate the association with an aerobiological airborne pollen monitoring study carried out during the studied period.

## Materials and methods

### Study area

Qatar is a peninsula that occupies an area of more than 11,000 km^2^ and has a coastline of 900 km in length. It lies between latitude 24° 27′ and 26° 10′ and longitude 50° 45′ and 51° 40′, connected to Saudi Arabia in the south, and bordered by a semi-enclosed sea, the Persian Gulf, characterized by hyper-salinity [17]. Geographically, it is a flat, rocky, and arid desert, with dunes being the predominant features in the south. As a subtropical desert, Qatar is hot and has dry weather, the annual rainfall being about 81 mm, as average, and the mean maximum temperature is 31 °C, although the maximum temperature could be beyond 47 °C [18]. Doha is the capital of the state of Qatar, located on the eastern Qatari coastal line at 25° 17′ 12″ N, 51° 32′ 0″ E. It is the most urbanized and populous city in the state, with a population of 2,641,669 in 2017 [19]. The main habitat and associated vegetation in the northern part of Qatar are characterized by numerous natural depressions of various sizes ‘rodat’, many of which have much deeper and richer soils. The type of soil present usually supports more moisture and organic matters than other parts of the desert and has, therefore, been converted to farmland. The vegetation is composed of a permanent framework of perennial and ephemeral plants that appear after rains. Desert plants are thinly scattered as they compete for the small amount of water available. The vegetation type usually ranges from trees and shrubs to grasses and herbs, commonly *Acacia* spp., *Prosopis juliflora*, *Ziziphus nummularia*, and *Lycium shawii* [20].

### Airborne pollen monitoring in the atmosphere of Qatar

Airborne pollen monitoring was carried out using two Hirst type seven-day recording volumetric spore traps (VPPS 2000 Lanzoni), from 8 May 2017 to 7 May 2019, representing two subsequent years. Two traps were placed on the roof of Building 310, Hamad Medical City, Doha (east Qatar) and Al Khor Hospital, Al Khor (north Qatar), at 15 m above ground level. Both traps were fixed on the corner of the rooftop of the selected buildings, 1 m above the roof. There were no adjacent higher buildings or air conditioners to cause airflow obstruction. The selection of these two areas was based on the surrounding vegetation and the presence of no physical barriers. The monitoring sampler operates on the principle of impaction through suction, with a 2 mm clockwise movement on the drum each hour (airflow rate was 10 L/min), and thus, the concentration of airborne particles could be calculated hourly (pollen grain/m^3^). The traps have a pump with a mechanism to suck a constant flow of 10 L per minutes and a regular check of the flow by an air flow controller is done weekly when the drum is changed. A recording tape, coated with a special type of silicone fluid as an adhesive substance, was collected weekly. Preparation, mounting, and counting of microscopic slides were done according to the standard protocol proposed by the Spanish Aerobiology Network; the REA [21], and the minimum requirements from the European Society of Aerobiology (ESA) [22]. Pollen concentration were expressed as pollen grain per cubic meter of air per day. Pollen from individual species of each family such as Poaceae and Amaranthaceae families were not differentiated in the counts under microscopy. In botanical classification, the Amaranthaceae family is a merger of the Chenopodiaceae and Amaranthaceae families, based on molecular systematics [30]. However, the family as a whole share a number of pollen-grain morphological features that render its members indistinguishable under light microscopy: grains are spheroidal, with a polypantoporate aperture arrangement. These pollen have morphological similarity and, with very few exceptions, cross-react [23]. The Annual Pollen Integral (APIn) expressed as Pollen * day/m^3^ is the sum of the daily mean pollen concentration over the given period [24]. The APIn of the principal allergenic pollen for each sampling area was taken into consideration to provide some estimation of the overall exposure to allergenic pollen.

### Detection of skin allergic sensitization to pollen extracts in asthmatic patients

The study was approved by the Ethical Committee of the Hamad Medical Corporation, Doha, Qatar (MRC#16150/16). All clinical investigations were conducted according to the principles expressed in the 1964 Helsinki declaration and its recent amendments. Written informed consent was obtained from all the participants in the study. Between May 2017 and May 2019, all patients referred for skin prick testing for inhaled allergens from the adult allergy and immunology clinics of the Hamad General Hospital were included.

Standardized allergen extracts of nine allergens from Allergopharma (GREER^®^ Extracts™, Greer Laboratories, Inc or AllerMed, San Diego, USA), including tree, grass, and weed pollen were used. The corresponding allergens were from the Poaceae family: Bermuda grass (*Cynodon dactylon*), seven grasses mix (Kentucky Blue (*Poa pratensis*), Meadow Fescue (*Festuca pratensis*), Orchard (*Dactylis glomerata*), Ryegrass (*Lolium* spp.), Redtop (*Agrostis gigantean*), Sweet Vernal (*Anthoxanthum odoratum*), Timothy (*Phleum pretense*), Dandelion, Fabaceae: Mesquite tree (*Prosopis* sp.), Alfalfa, Amaranthaceae: *Chenopodium album*, Rough pigweed (*Amaranthus retroflexus*), Russian thistle (*Salsola* sp.), and Compositae: Mugwort (*Artemisia* sp.). Readings were performed by a physician 15 min after the allergens over the skin were pricked. After that, in order to evaluate the results, the diameter of the wheal was measured. A positive reaction was considered when the wheal was at least 3 mm larger than the induced concurrent negative control skin testing, and the positive control of histamine reaction was at least 3 mm in diameter or the allergen reaction was greater than or equal to 50% of the histamine reaction [25, 26].

### Symptoms questionnaire

Symptom questionnaires were conducted for patients with a positive skin prick test to pollen extracts. The brief questionnaire was composed of specific questions to identify basic information of patients’ symptoms suggesting the diagnosis of asthma, allergic rhinitis, and atopic dermatitis. The questionnaire was adapted from the International Study of Asthma and Allergies in Childhood (ISAAC) with adding questions about the diagnosis made by the treating physician and frequency of exacerbation occurring over the previous 12 months of the year [2, 27].

### Statistical analysis

The analyses were performed, using data obtained from May 2017 to May 2019. The results were presented as means with a range for normally distributed data and frequency (number and percentage) of participants as appropriate. Correlations between symptoms and airborne pollen concentrations were demonstrated using the Spearman correlation test. Data were analyzed using the Statistical Package for Social Sciences software (SPSS Chicago IL, USA) for Windows, Version 21.0. A *P *≤ 0.05 was considered statistically significant.

## Results

### Aerobiological pollen records

During the study period (May 2017–May 2019), low pollen concentrations were recorded in both sampling sites due to the low-density cover vegetation typical of arid area. An APIn of 1977 was detected in the atmosphere of Qatar, from which 1359 corresponded to Al Khor and 618 to Doha. The pollen belonged to 25 different taxa. In both years, pollen from weed plants had the principal contribution to the airborne pollen spectrum (Doha, 52.5%; Al Khor, 71.0%), followed by trees (Doha, 21.4%; Al Khor, 14.6%), and grass (Doha, 21.7%; Al Khor, 11.6%). The most common taxa detected, which made up the total amount of pollen recorded in the atmosphere of Qatar, proceeded from native plants, such as Amaranthaceae, Poaceae, *Prosopis* (*Mesquite is a common name for several plants in the genus of Prosopis*), *Ziziphus*, *Dandelion (one of the species included in Asteraceae family*) *as well as introduced plants such as Cupressaceae, Casuarina, Conocarpus and Alfalfa* (*one of the species included in Fabaceae family*). Detailed percentages of the annual contribution are provided in Table 1.

**Table 1:** The Annual Pollen Integral (APIn) (day/m^3^) recorded in the station of Doha and Al Khor during the period (May 2017–May 2019)

Taxon	Aerobiological station		Aerobiological station	
	Doha		Al Khor	
	Σ APIn	%	Σ APIn	%
Weeds				
Amaranthaceae	240	39.2	800	58.9
Apiaceae	1	0.16	7	0.51
Artemisia	11	1.80	30	2.2
Asteraceae	5	0.82	2	0.15
Brassicaceae	16	2.6	28	2.05
Cyperaceae	20	2.2	38	2.79
Ephedra	1	0.16	6	0.5
Euphorbia	1	0.16	0	0
Plantago	22	3.59	35	2.57
Ricinus	4	0.65	8	0.59
Rumex	4	0.65	6	0.73
Urticaceae	7	1.14	7	0.51
Arnebia	0	0	1	0.07
Grass				
(Poaceae)	132	21.7	158	11.6
Trees				
Casuarina	14	2.29	16	1.17
Conocarpus	12	1.96	47	3.45
Cupressaceae	12	1.96	15	1.10
Myrtaceae	1	0.16	1	0.07
Meliaceae	4	0.65	0	0
Olea	7	1.14	12	0.88
Arecaceae	28	4.58	50	3.67
Prosopis	32	5.23	39	2.86
Ziziphus	8	1.31	4	0.29
Parkinsonia	0	0	4	0.29
Fabaceae	9	1.47	4	0.29
Indetermined	27	4.41	41	3.01

*APIn* Annual Pollen Integral

**Fig. 1:**
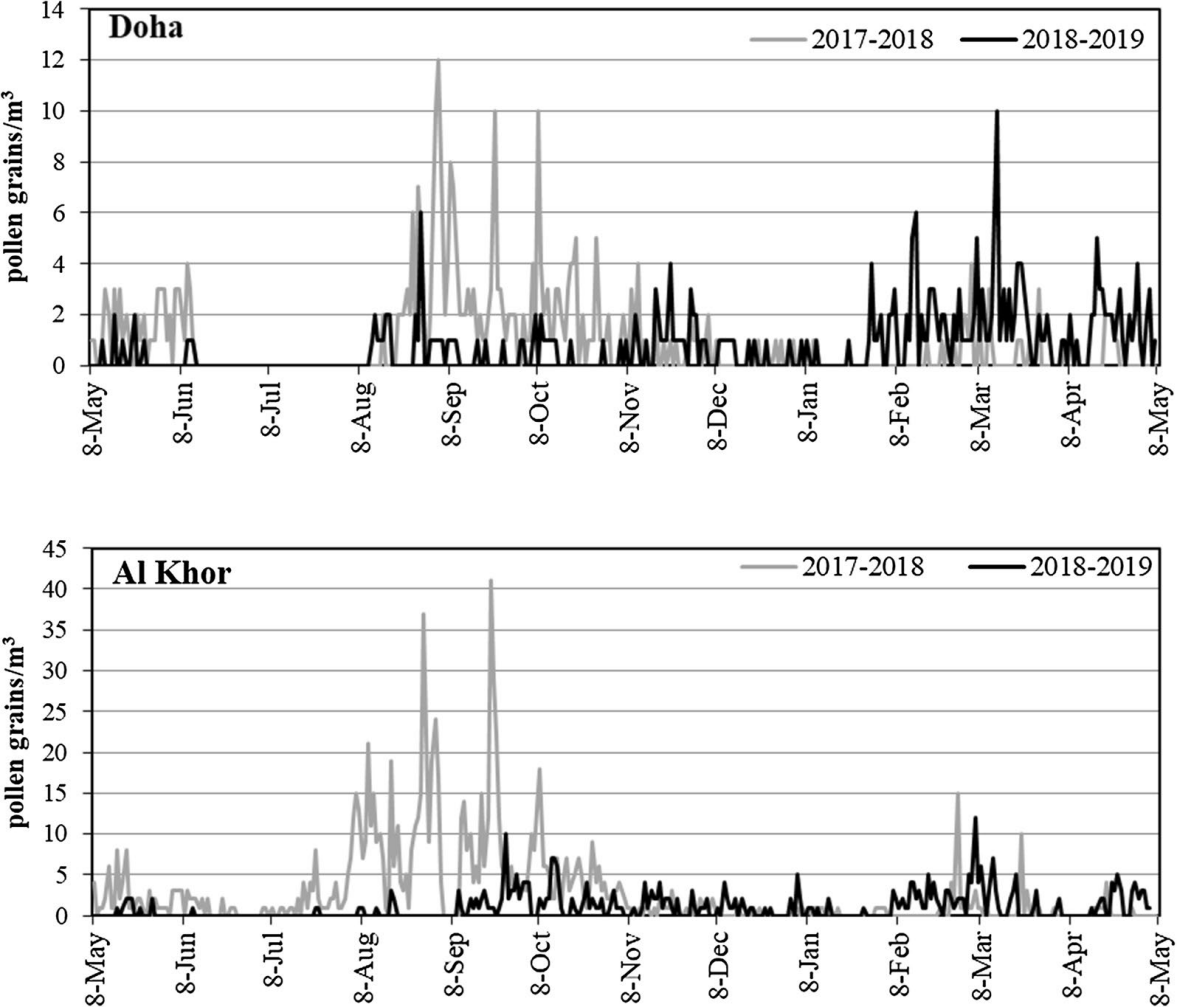
Seasonal variation of the daily mean total count (pollen grain/m^3^) of pollen in the sampling sites. Two sampling stations in two different cities recorded pollens over 2 years in the atmosphere of Qatar. The seasonal peak was observed in the periods between August to October, and February to March. The maximum concentration of pollen was 40 pollen grains/m^3^

When seasonal distributions of total pollen are taken into account, considerable differences in daily mean pollen concentrations could be observed between the two studied years, being higher in stations of Al Khor. Different peaks were found during different parts of the year, representing the periods of pollen emission of a group of different taxa that make up the pollen spectrum. Moreover, in the first year of the study, the maximum pollen concentrations were recorded between August and October, while the period from February–April had the highest pollen concentrations in the second year. In the atmosphere of Qatar, the highest monthly pollen concentration was detected in September 2017; with a maximum daily pollen peak of 41 pollen grain/m^3^ recorded at Al Khor station (Fig. 1).

**Fig. 2:**
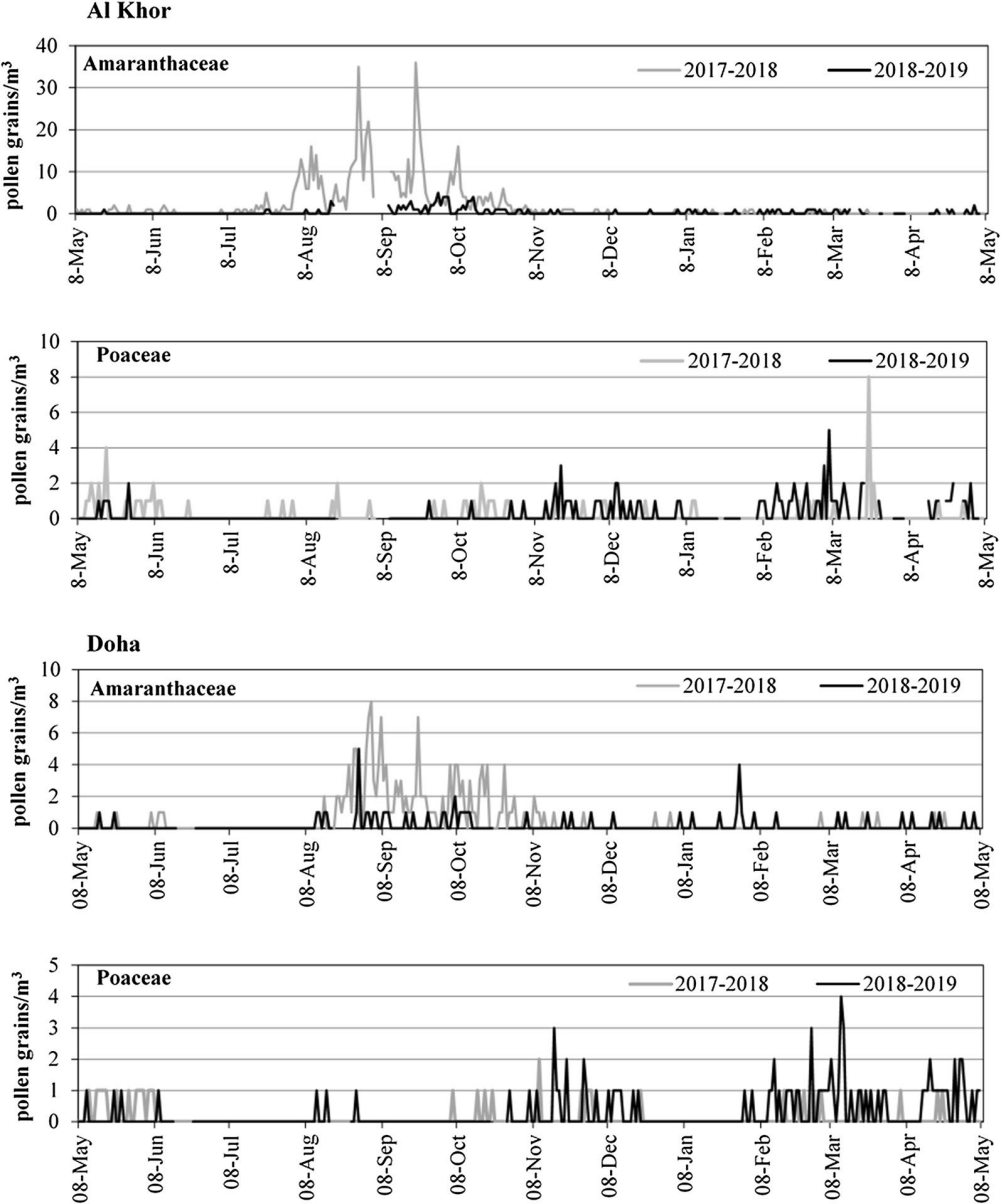
Seasonal variation of the daily mean concentration (pollen grain/m^3^) of the two main pollen (Amaranthaceae and Poaceae) in the aerobiological sampling sites Doha and Al Khor. Higher airborne pollen concentration was detected in the atmosphere of Qatar in the year 2017–2018

Regarding the dynamics of the two first taxa in terms of pollen concentration in the air, different patterns were observed in the two-yearly periods of study with great differences between both sampling sites. Higher pollen concentrations were recorded in Al Khor, while the lowest values were obtained in Doha. Amaranthaceae presented their highest pollen concentration during 2017–2018, and lower during 2018–2019. The highest pollen concentration was recorded from the first week of August to the end of October and 36 pollen grain/m^3^ was the peak value recorded in September 2017 (Al Khor station). Poaceae, on the other hand, showed their highest pollen concentration between the beginning of February to the end of March. Higher pollen concentration was obtained at both stations during the year (2018–2019), with a total of 39 pollens. Poaceae showed one peak value of eight pollen grain/m^3^ during March 2018 in Al Khor (Fig. 2).

### Demographic variables of patients

Among 1000 patients referred from the allergy and immunology clinics for SPT to inhaled allergens and fulfilled the criteria for enrolment from May 2017 to May 2019, a total of 940 patients (430 males and 510 females) with a mean (range) age of 30 (20–61) years, agreed to participate in this study. Qatari nationals comprised 43.7% of the study population, and the remaining were mainly nationals from other Arab (Egyptians, Syrians, and Lebanese), Asian (Indians, Bangladeshis, and Filipinos), and other countries (UK, Canada). Three-hundred and eighty-three (40.7%) tested negative to all allergens.

### Detection of skin allergic sensitization to pollen

The skin prick test reaction was positive in 557 patients, with a total of 839 (89.2%) inhaled allergens. Among these patients, 382 patients (68.6%) had polysensitization; 204 (21.7%) had positive sensitization to pollen, 324 (34.5%) to dust mite, 48 (5.1%) to fungal spores, and 263 (28%) to animal proteins (cat hair, cockroaches, and horses). Among the patients with sensitization to pollen, 120 were males (59.1%), and 84 were females (40.9%), with a mean (range) age of 37 (31–40) years (Table 2).

**Table 2:** Demographic variables of patients with positive SPT to pollen (n = 204)

Demographic variable	No	%
Age (years)		
< 20	27	13.3
21–30	46	22.7
31–40	48	23.2
41–50	40	19.7
51–60	34	16.7
> 61	9	4.4
Sex		
Male	120	59.1
Female	84	40.9
Nationality		
Qatari	89	43.8
Non-Qatari	115	56.2

Analyzes of the frequency of sensitization to pollen allergens revealed the highest frequency of positivity for allergens of the Amaranthaceae pollen (66.2%), followed by the Poaceae (55.8%). The detailed frequency and percentages of positive skin reactions for all studied pollen allergens are summarized in Table 3.

**Table 3:** Results of the frequency of sensitizing to pollen allergens (n = 204)

Positive to pollens	No	%
Chenopodium	135	66.2
Poaceae	114	55.9
Mesquite	37	18.1
Dandelion	22	10.8
Alfalfa	18	8.8

**Fig. 3:**
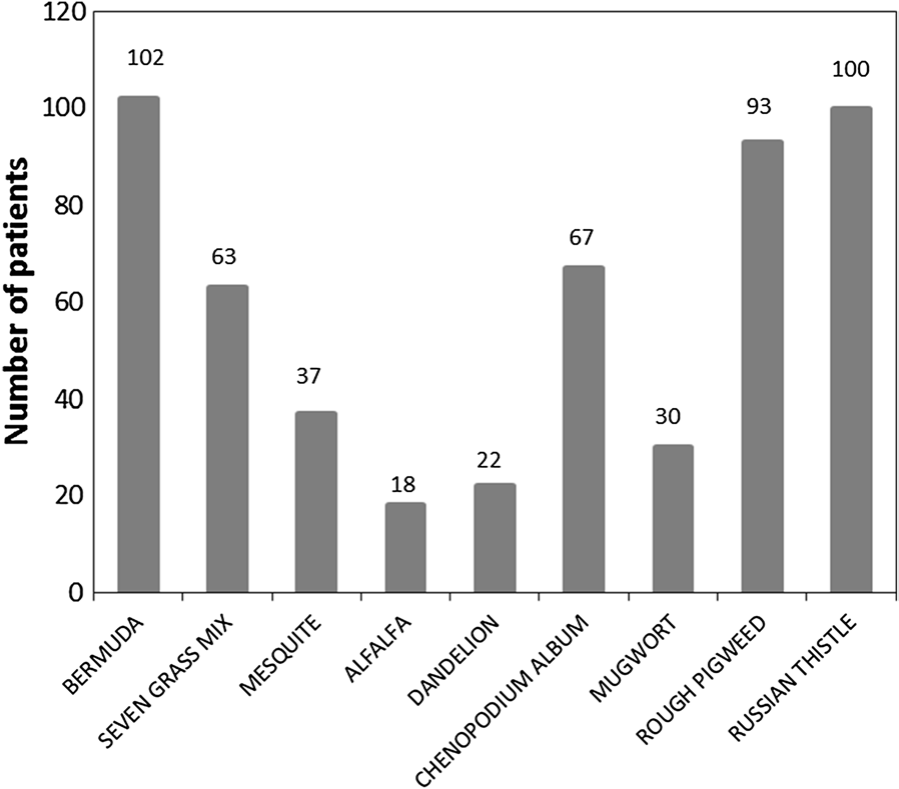
Frequency of sensitization to individual pollen allergens (n = 204). Some patients have more than one allergen

The frequencies of sensitization were Bermuda grass 102 (50%), Russian thistle 100 (49%), Rough pigweed 93 (45.6%), and *Chenopodium album* 67 (32.8%). The relative proportions of patients positive to specific pollen allergens are shown in Fig. 3.

### Correlation between symptoms and allergenicity

**Fig. 4:**
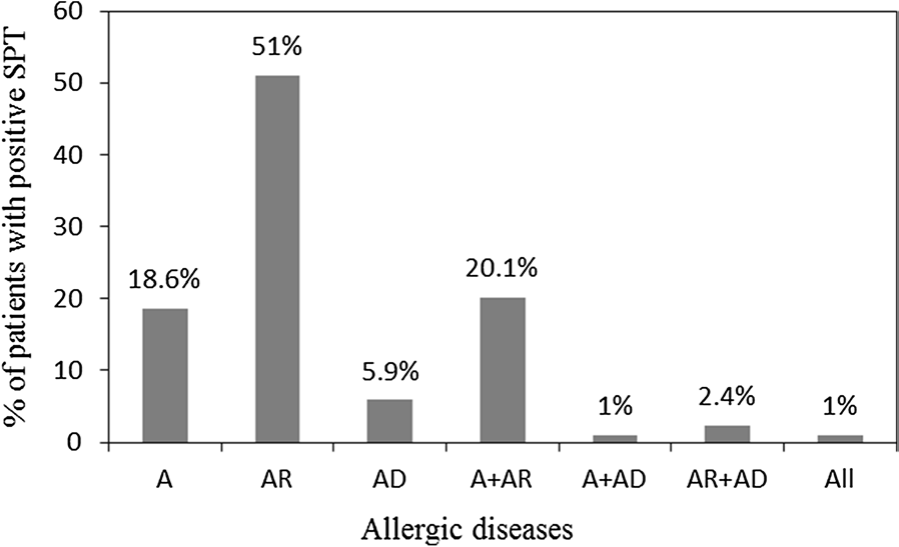
The distribution of allergic diseases among adult patients who attended the allergy clinics in Qatar during the study period (n = 557)

The symptoms questionnaire was performed for all the examined patients with positive SPT to pollen allergens to stratify the cohort into the disease type (asthma, allergic rhinitis, and atopic dermatitis). Each of the 204 patients had positive symptoms suggesting at least one allergic disease. A comparison was made between the percentage of patients with allergic diseases and those with positive sensation and showed that 38 (18.6%) patients had asthma, 104 (51%) allergic rhinitis, and 12 (5.9%) atopic dermatitis. Moreover, 50 (24.5%) had more than one disease. Detailed percentages are presented in Fig. 4.

The correlation between patients’ symptoms levels and sensitization to pollen allergens were analyzed using Spearman’s correlation tests. Significant correlations between sensitization levels to Amaranthaceae and the symptoms of asthma (r = 0.169, *P *= 0.016) and allergic rhinitis (r = 0.177, *P *= 0.012) were found. Moreover, positive correlation coefficients, but not reaching statistical significance, were noted in all of the common diseases associated with individual allergens (Table 4).

**Table 4:** Correlation coefficients (r) between the patients’ symptoms and pollen allergens in skin prick testing (n = 204)

Allergic diseases	Poaceae		Chenopodium		Mesquite		Alfalfa		Dandelion	
	*r*	*P*	*r*	*P*	*r*	*P*	*r*	*P*	*r*	*P*
Asthma	0.117	0.095	0.169*	0.016	0.047	0.503	0.125	0.075	0.043	0.544
Allergic Rhinitis	0.075	0.287	0.177*	0.012	0.094	0.180	0.016	0.817	0.097	0.167
Atopic Dermatitis	− 0.041	0.561	0.073	0.300	0.007	0.925	0.087	0.215	0.113	0.110

* (r) Correlation significance at *P *< 0.05

## Discussion

The present study is the first detailed report on the airborne pollen spectrum in the atmosphere of Qatar. As such, the study provided basic information aimed to examine the possibility that the degree of pollen exposure influences the prevalence of the symptoms of allergic rhinitis and asthma in Qatar, using volumetric sampling methods.

Pollen/aeroallergens constituted one of the essential groups of sensitizing allergens leading to allergic rhinitis and asthma in our region with as many as 21.7% out of the 204 were sensitized to at least one pollen type. These findings were consistent with other neighborhood countries with similar hot and desert climate and where airborne pollen are considered major allergens [28, 29].

The aerobiological air monitoring was initiated as a pioneer project in the peninsula of Qatar, using Lanzoni Volumetric samplers for recording the airborne pollen spectrum composition and seasonal variation, in addition to establishing a pollen level forecast in Qatar. However, more years are needed for air pollen monitoring because of the high variability in yearly behavior.

The pollen detected in the samplers’ stations reflected the natural distribution and the abundance of the surrounding anemophilous vegetation at these sampling sites. Precisely, plants of the Amaranthaceae, are predominantly spontaneous perennial weeds growing on salt-enriched soils, producing relatively high local concentrations of pollen and being able to induce pollinosis, since this pollen type has been identified as the most abundant in our samplings in the study areas. Recently, the Amaranthaceae family has been extended to include the Chenopodiaceae family based on morphological and phylogenetic analyses [30]. The species of the genus Amaranthus and Chenopodium shares a number of pollen-grain morphological features that render its members indistinguishable under light microscopy, as is the case of the Poaceae species [23]. Due to this substantial similarity, all the genera are considered of the same pollen type during the pollen counting.

Amaranthaceae and Poaceae (grass) were the most common airborne pollen types detected in the aerobiological study carried out in Qatar. This finding is in accordance with the data obtained from other studies carried out worldwide, which rank Poaceae and Amaranthaceae among the major aeroallergens causing allergy symptoms, especially in North America and Europe [3, 4].

The annual pattern of variation in pollen concentration was very similar at both stations in the 2 years, with maximum daily mean concentrations recorded during August for the Amaranthaceae pollen (2017–2018) and March for the pollen of Poaceae (2018–2019). A similar pattern was found in other countries in the Middle East, such as Kuwait [10, 11] and Saudi Arabia [12, 13]. These periods may represent the pollen seasons in Qatar. Generally, grass and Amaranthaceae pollen predominate in the spring period (May–June) in Southern Europe [31] and North America [32]. However, Amaranthaceae and Poaceae showed different patterns with other smaller peaks observed in late January (Amaranthaceae) and November to December (Poaceae), during the winter and cold seasons of Qatar.

A possible explanation for this observation is that in the studied area, the weather is characterized by two periods; the cold season (October to March), when the temperature is generally under 25 °C and the warm season (April to September, when the temperature ranges from 26–44 °C on most of the days) [33], thus favoring the presence of different species with different ecological requirements. The case is especially noted for species adapted to salty soils. For example, many species from the Amaranthaceae are present in Qatar such as *Salsola*, *Halopeplis*, *Arthrocnemum*, or *Halocnemum*, with *Salsola* being one of the principal causes of pollinosis in arid lands [34]. Moreover, Poaceae and Amaranthaceae are the most diverse families, including a large number of species in Qatar, growing in urban and natural desert areas in Qatar [20], with different flowering seasons, depending on the weather conditions.

Based on the result of the skin prick tests carried out on patients with allergic diseases, the Amaranthaceae species remained a dominant cause of respiratory allergy. Due to their seasonality, and because these plants are wind-pollinated, their pollen are among the crucial causes of summer allergies in the Mediterranean countries [34], as well as in areas with desert climates [28, 29].

The grasses are the second abundant airborne pollen in the atmosphere of Qatar. Worldwide, at least 40% of allergic patients are sensitized to grass pollen allergens [35]. In Europe, 20% of the population is affected by grass pollen allergy [4], compared to 15% in the United States [36].

In Qatar, Bermuda grass is common and found mostly in the residential, cultivated areas, and along roadsides. It is often grown as lawns but grows abundantly on its own. Our data showed that 114 (55.8%) patients had sensitization to grass pollen. Bermuda grass (102, 50%) and the seven grass mix (63, 30.9%) were the most common among the sensitizing agents. Similar results were found in other countries such as Kuwait [11], North America [37], and Australia [38].

In this study, the extracts of other outdoor pollen types, with limited contribution to the total airborne pollen concentrations, showed a positive SPT among the allergenic patients. For example, Mesquite trees are wind-pollinated and endemic species in Qatar. The air monitoring samplers recorded lower daily mean concentrations without any specific peak during the 2 years of the study; however, the SPT revealed 37 (18.1%) positive results. These data may not indicate that this pollen type is not allergenic as reported in other Asian countries [28, 39] and USA [40], but it could be underestimated due to the pollen sources which are far from the sampling sites.

The result of our study showed that there is a highly significant association (*P *< 0.05) between the Amaranthaceae extract SPT results and the symptoms of asthma and allergic rhinitis. Similar results were reported in countries such as Spain [34] and Kuwait [28, 29] supporting the antigenic properties of Amaranthaceae pollen in the sensitization of patients with atopic asthma and allergic rhinitis.

The statistical correlation analysis carried out between allergic diseases and the different airborne pollen types tested was not very relevant except in the association between Amaranthaceae pollen and asthma and allergic rhinitis symptoms among the patients recruited for the study. It may be that pollen concentration from a single sampling site does not cover the geographical area of influence in the patients. Moreover, the lower correlations can be due to low pollen concentrations in this area.

Standardized aeroallergen extracts were utilized in the skin prick test in the current study. The panel of plant allergens belonged to closely related species with significant cross-reactivity. The degree of cross-reactivity among pollen extracts from the Chenopodiaceae species and those of the taxonomically related Amaranthaceae family has been demonstrated [41]. The different Chenopodiaceae and Amaranthaceae species contain common Immunoglobulin E (IgE)-binding antigens; however, specific allergen characteristics of each species are considered important in allergenicity. Cross-reactivity can also be displayed by allergens from not closely related species, such as the *Chenopodium album* pollen that shares common reactive proteins with Poaceae, Betulaceae, and Oleaceae [42].

Some limitations in our study must be considered in the interpretation of the findings, including the duration of the aerobiological sampling (2 years). Based on differences recorded over several years of aerobiological studies in different countries, long-term samplings are recommended to better establish daily and annual patterns for the different pollen types in a locality [31, 32]. However, 2 years were considered sufficient to provide preliminary data that could help healthcare providers to identify the commonest pollen and provide the treatment approach and the needed immunotherapy [13]. Another limitation is the number of samplers that may be needed for covering the whole peninsula of Qatar. In our study, two aerobiological samplers were used and placed in two cities with native and naturalized plants of Qatar and a high population, particularly, Doha city that has a population of around 800,000 inhabitants. It is known that more detailed analysis of pollen concentration with regard to local pollen sources and clinical data are needed in order to evaluate the correlation among different pollen conditions and allergy symptoms [5]. Furthermore, dust in the air of Qatar could profoundly hinder pollen trapping due to the inorganic particles of sand that could compete with pollen for the space in the adhesive tape used for pollen collecting. Large quantities of dust particles were observed in the collected tapes. So, pollen concentrations could be underestimated.

Finally, our findings further support the conclusion that this study is an important step to establish a more comprehensive aerobiology network throughout the Arab Gulf region.

## Conclusion

Aerobiological monitoring can be an essential tool for identifying the atmospheric pollen concentration across the variable seasons, improving the quality of the urban environment and protecting pollen-allergy sufferers by using prevention measures and allergen specific immunotherapy. The data presented is limited in time; however, pollen can be one of the main sensitizing allergens among patients with allergic rhinitis and asthma in Qatar, along with other known indoor allergens and may contribute to the pathogenesis of allergic respiratory diseases. There are no previous studies in the same region to make any comparison between environmental factors, such as social practices, increasing mobilization, and introduction of new potentially allergen-producing plant species in urban areas for greening, the causation of allergic diseases and our results. The result of this study is mostly consistent with the result of other studies in other parts of the world, with some variations due to differences in environmental conditions and can be a foundation for new research areas in the desert climate.

## Data Availability

The datasets used and/or analysed during the current study are available from the corresponding author on reasonable request.

## Abbreviations

SPT: Skin prick test
APIn: Annual Pollen Integral
REA: Spanish Aerobiological Network
